# Epidemiological surveillance of the HIV/AIDS complex through the analysis of trends in the incidence of Kaposi's sarcoma in Cali, Colombia

**Published:** 2012-12-30

**Authors:** Alejandra Saldarriaga-Cantillo, Luis Eduardo Bravo, Óscar Londoño, Luz Stella García, Paola Collazos

**Affiliations:** a Cancer Registry of Cali. Departament of Pathology. Universidad del Valle, Cali, Colombia. E-mail: alejandrasaldarriaga20@yahoo.com,; b Secretaria de Salud Publica Municipal de Cali.

**Keywords:** Kaposi sarcoma, HIV-AIDS, antiretroviral therapy, TAAR

## Abstract

**Introduction:**

The Kaposi's sarcoma (KS) incidence has markedly changed in the general population since the onset of the AIDS epidemic in the eighties and after the introduction of the Highly Active Antiretroviral Therapy (HAART) in the nineties.

**Objective:**

To investigate incidence rate trends for Kaposi's sarcoma before and during the (HIV/AIDS) epidemic in Cali, Colombia.

**Methods:**

Exploratory ecological study that included all Kaposi's sarcoma cases identified by the Cali Cancer Registry from 1962-2007, and 12,887 cases of HIV/AIDS recorded in the Municipal Health Secretariat of Cali between 1986 and 2010. The joinpoint regression model was used to conduct the incidence rate analyses between the years 1962 and 2010.

**Results:**

A total of 349 KS cases were identified during the study period. Only 5.3% of the cases (n=20) were diagnosed in the pre-epidemic era (1963-1987), of these, 35% were women, and 90% of the tumors were located on the skin. In contrast, 94.7% of KS cases (n=329) were discovered after the emergence of HIV-AIDS. There was a significant decrease in the proportion of women (10.9%, *p* <0.001) and an increase in the frequency of tumors with an extra-cutaneous location (19.1%, *p* <0.01) compared to those cases diagnosed in the pre-epidemic era. Notification rates of HIV/AIDS have decreased since 2002 in both genders but KS incidence rates have decreased since 2004 in men only.

**Conclusion:**

The downward trend in the incidence of these diseases may be associated with factors that prevent the transmission of HIV infection or limit the spread of HIV in the community. Cancer registries represent a resource for timely, population-based surveil-lance of HIV-associated malignancies in Cali, Colombia.

## Abstract (es)

**Introducción:**

La incidencia del sarcoma de Kaposi ha variado de manera significativa con la aparición de la epidemia de la infección por el virus de la inmunodeficiencia humana/síndrome de inmuno-deficiencia adquirida (VIH/SIDA) en la década de los ochenta y con la aplicación de la terapia antiretroviral altamente activa en la década de los noventa.

**Objetivo:**

Examinar los cambios en la tendencia de la incidencia del sarcoma de Kaposi antes y durante la epidemia de la infección (VIH/SIDA) en Cali, Colombia.

**Métodos:**

Estudio ecológico exploratorio que incluyó todos los casos del sarcoma identificados por el Registro poblacional de cáncer de Cali, en el período 1962 al 2007; y los 12.887 casos de VIH/SIDA registrados en la Secretaría de Salud Pública Municipal de Cali, entre 1986 y 2010. Para estudiar los cambios en la tendencia de las tasas de incidencia y notificación se utilizó el análisis de regresión de Joinpoint.

**Resultados:**

Durante el período del estudio se identificaron 349 casos de SK. Sólo el 5.4% (n=20) se diagnosticó en la era pre-epidémica (1963-1987), de ellos, 35% ocurrieron en mujeres y 90% de los tumores se localizó en la piel. En contraste, el 94.7% (n=329) de los casos de SK fue descubierto después de la emergencia de HIV-SIDA, con una disminución significativa en la proporción de mujeres afectadas (10.9%, *p* <0.001) y aumentó en la frecuencia de tumores con localización extra-cutánea (19.1%. (*p* <0.01). Desde el año 2002, se evidenció disminución del riesgo VIH/SIDA en ambos sexos y desde el año 2004 disminución del riesgo de sarcoma de Kaposi sólo en los hombres.

**Conclusión:**

La tendencia decreciente en las tasas de incidencia de estas enfermedades, puede estar asociada con factores que previenen la infección del HIV o que restringen su desarrollo. Los Registros de cáncer representan un recurso para la vigilancia epidemiológica poblacional oportuna de las neoplasias malignas asociadas con el HIV.

## Introduction

Persons infected with HIV/AIDS present an increased risk of developing some type of cancer during the course of the disease, which can be estimated with a percentage rate somewhere between 30 to 40%. The risk is greater for certain types of cancer, termed AIDS-defining, such as Kaposi's sarcoma (KS), non-Hodgkin lymphoma (NHL), and cervical uterine cancer.^1^

The relationship between HIV/AIDS and certain cancers is not fully understood, but the link is likely related to the immunosuppressant state and the high prevalence of risk factors for cancer development, including, among others, tobacco usage and ontogenic viral coinfection.

KS is recognized as one of the most common cancers in persons with HIV infection. Its incidence is 20,000 times greater than that of the general population and 300 times greater than that found in patients with other causes of immunosupression.^2^ The behavior of KS has been greatly influenced by the HIV epidemic trend in the population and by the measures that have been taken for its control.^1^ ^,^ ^3^ For example, a significant increase in the KS incidence was evidenced during the 1980s by the spread of the HIV infection and from 1996, this incidence was reduced with the introduction of highly active antiretroviral therapy (HAART, is the English acronym).^4^ These findings come from longitudinal population studies conducted in the U.S. and Europe after the introduction of HAART. Therefore, the results of these studies cannot be easily extrapolated to the current KS incidence trends in the general population of Latin America.

The effect of HAART across the cancer definition spectrum remains unknown in Cali, Colombia. This article describes changes in the incidence of KS before and during the HIV/AIDS epidemic in the period between 1962 and 2010.

## Materials and Methods

### Population Studied and Location

The Population-based Cancer Registry in Cali (RPCC) has operated continuously since 1962. The registration area is 110 km² and is located in the city's urban area. Cali is the third largest city in Colombia and is the capital of the Department of Valle del Cauca, one of the 32 departments in Colombia. After the 2005 census the population of Cali was 2,030,000. Most of the population is mestizo with a minority of blacks and whites. The population distribution shows more than 50% are immigrants from other parts of the country and a small percentage are from other countries^9^.

The RPCC database has information on the cancer case incidence in Cali since 1962. It is the diagnostic base for more than 180,000 new cancer cases in the city's urban area due to the active and ongoing data search of all sources. The RPCC methodology and case definitions have been previously described.^5^ For quality assurance, the RPCC uses procedures promoted by the IARC to ensure the validity, completeness, accuracy and comparability of cancer registry information. The procedures were reviewed and approved by the Institutional Ethics Committee of the School of Health at the Universidad del Valle. Based on database records, the KS trend for the city of Cali, during the period from 1962 to 2007, was determined.

### Collection and consolidation of HIV/AIDS records

The HIV/AIDS registries, that are part of this study, are confirmed cases which were collected by notification or detected by a search of Cali residents. Notification is a systematic process of collecting and capturing information on confirmed cases of HIV/ AIDS and deaths from AIDS through the INS 850 Code of the National Public Health Surveillance System, SIVIGILA.

Most institutions report cases by means of SIVIGILA. The software stores the data collected on each entry and generates a file that is emailed to the Epidemiological Surveillance Office of the Municipal Secretariat of Health in Cali. Institutions without an informational infrastructure physically send a data card by courier or by fax. After a review and coding process the information is entered into SIVIGILA.

The active search for cases, by reviewing death certificates, complemented the information obtained by notification and allowed for updating the vital status in the database of Persons Living with HIV (PLHIV), detecting cases of death from HIV/AIDS that had not been previously reported, identifying in the general mortality database the sequence of events underlying the cause of death in persons who were already made note of but were recorded as deaths from a cause other than HIV/AIDS.

The confirmed case data cards are processed in all health institutions that attend HIV patients or in the clinical laboratories that confirm the diagnosis. The interagency management of some patients makes it possible for the same case to be reported by differing institutions. Many of the records obtained had inconsistencies in one or more variables and required a careful screening process to validate the information. The pairing of the patients with HIV/AIDS databases with the general mortality data base optimized the screening and editing of information and allowed for updating the vital status of each person and allowed for making any needed corrections to the information concerning the name, sex , age, date of birth, and identity card number.

After making any needed adjustments to the identification data for each record and possibly correcting other variables, new cases were defined and distinguished from duplicates previously reported. In prevalent cases, the condition of the patient was updated (e.g. change made to AIDS or changed to death). When missing or inconsistent information was presented for important variables in some records, it became a requirement that the institution report on it. In the majority of cases the corrections, completions or missing data recoveries were obtained.

For the year 2010, 8% of the notifications of HIV/AIDS cases corresponded to cases previously reported during earlier stages of the infection. This proportion could reach up to 15% through repetition of the notification or because of earlier correspondence for a change in the state of the infection. The repeated notifications may indicate that the level of reporting in Cali is high. While underreporting in other Colombian cities (Bogotá, Medellín and the country in general) can exceed 50%, in Cali underreporting is estimated at no more than 20%.

### Methodology

This exploratory ecological study describes the incidence rate trends for KS during the period 1962 - 2007; the trend in the reporting rates for HIV/AIDS complex from 1986 to 2010, and how these factors mutually affected each other from the moment of their temporal coincidence.

Based on the sarcoma cases identified by the RPCC, classifications with the 9140 CIE-0-3 code were made, including cases of HIV/AIDS recorded by the Municipal Health Secretariat of Cali that were coded according to the International Classification of Diseases 9th Edition (ICD-9) for the period 1986-1996, and according to the 10th Edition (ICD-10) for the period of 1997-2010.

The study included the registries of KS and HIV/AIDS for persons of all ages categorized into age groups according to the family life cycle, ^6^regardless of gender and residence in Cali, and contained within the databases previously noted. Duplicate and incomplete information from the registries was discarded.

### Statistical Analysis

For both factors, estimations were made for the crude rates, and for specific rates by sex and age groups (<20, 20-39, 40-59, ≥ 60 years). The standardized rates by age were calculated using the direct standardization method which uses the standard world population as reference. All rates were expressed as per hundred thousand persons by year (p:y). The composition of the population of men and women, in Cali, was obtained from the census and official standards published by the National Administrative Department of Statistics (DANE) for the years 1973, 1985, 1993 and 2005. Each year's population size was estimated by geometric interpolation between the periods contained in the analysis. To determine the composition of the population for each year for the 2004-2010 period a geometric extrapolation was performed based on the 2005 census. Estimates of the rates and their standard errors were performed using the Stata^7^ program.

To describe the sex-specific standardized rate trends for each period studied, we conducted a regression analysis using the Joinpoint Software, version 3.5.4.^8^ ^9^

The objective of the approach was to identify when possible significant change points occurred in the lineal logarithm trend. In the final model, each Joinpoint indicates a statistically significant change in the trend and an annual percentage change (APC). Each of these trends was calculated by using a generalized linear model while assuming a Poisson distribution. Standardized annual rates and their standard errors were used for the estimation of these models. It was considered that there was an increase or decrease when the slope (coefficient) of the trend showed statistically significant differences (two-tailed, p <0.05). If this were not the case, the trend was considered stable.

## Results

### HIV/AIDS Complex

Notification of the first HIV case in Cali was made in 1985 involving a 56-year-old man. There were no additional cases during that year. During the period 1986-2010 12,887 cases were registered and recorded by the Municipal Health Secretariat of Cali, of these 74.9% were males. The average age disease onset in women was 32.5 years, CI 95% (31.9-32.9). This was significantly younger than the average age observed in men, i.e. 35.2 years, CI 95% (34.9-35.5).

The proportion of women affected by the HIV/AIDS complex increased from 8% (31/388) in the 1986-1990 period to 29.4% (1242/4335) during 2006-2010, *p* <0.001. The HIV/AIDS incidence rate ratio standardized by age (male: female) went from 12.6 in 1986-1990 to 2.6 in 2006-2010 (Table 1).

**Table 1.**
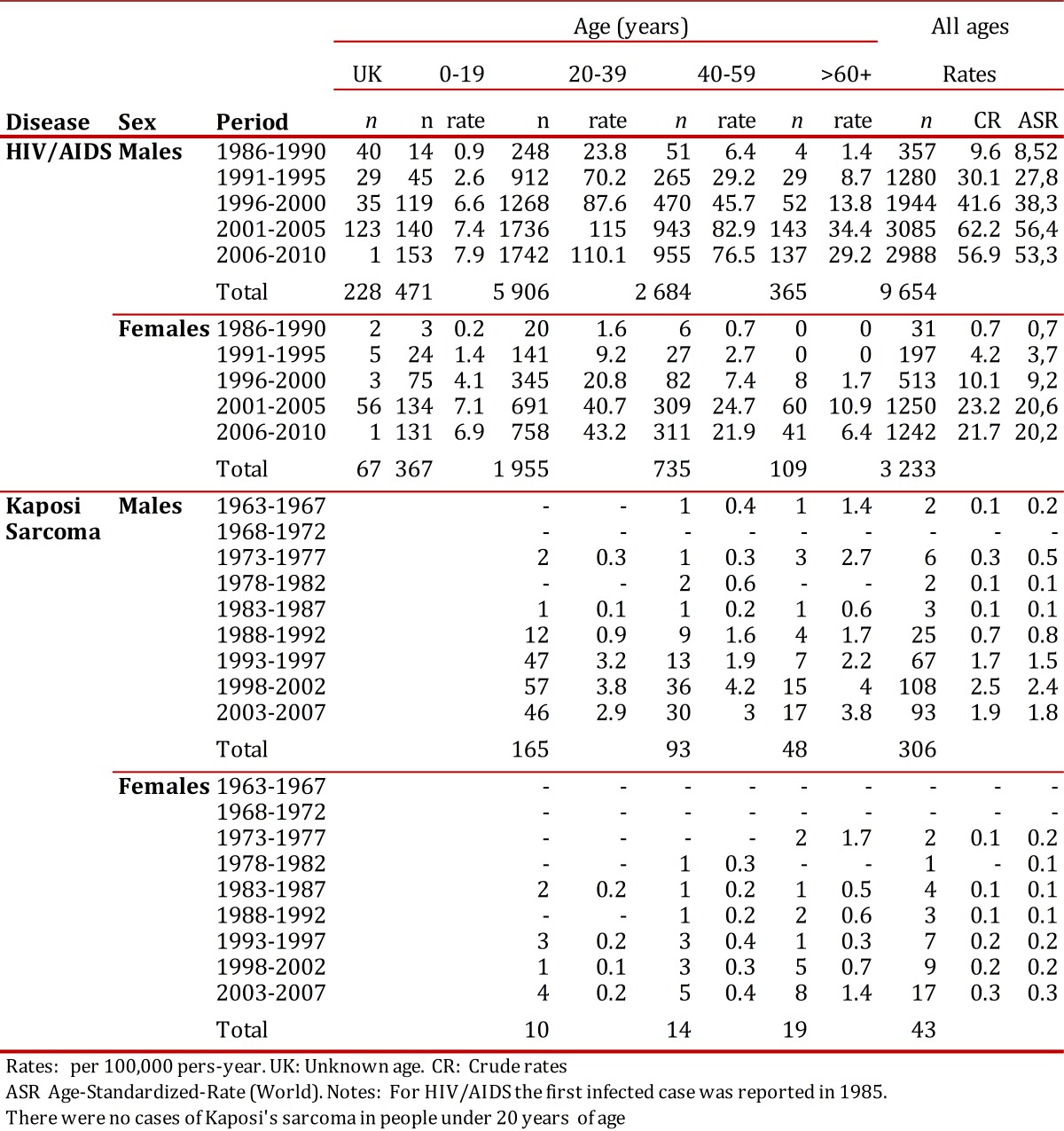
Cali, Colombia. Number of cases, Age-specific rates, Crude and Age-standardized-rates (World) for Kaposi Sarcoma and HIV/AIDS by sex, from 1962 to 2010.

### Reporting rates for the HIV/AIDS complex

**Figure 1:**
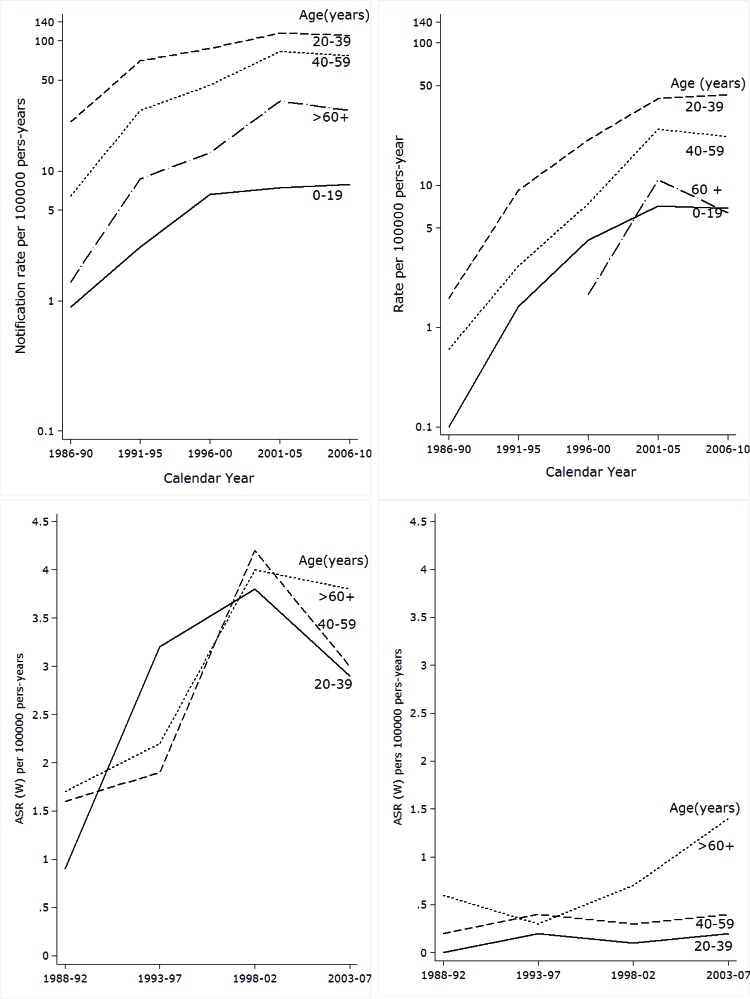
Cali, Colombia. Age-Specific trends in Kaposi's sarcoma Incidence rates from 1962 to 2010 and Age-specific Trends in HIV/AIDS notification rates trough 1985-2010.

Specific notification rates by age increased in all age groups until the period of 2001-2005. There was a clear effect by period. Starting from this five-year period there was a reduced risk of HIV-AIDS in most age groups for both men and women (Table 1 and Figure 1). In all periods studied and for both sexes the difference between notification rates by age and the crude rate was less than 10%.

During all periods evaluated, it was found that men and women between the ages of 20-39 years had a higher risk of HIV/AIDS. The specific reporting rate in males reached its peak in the 2001-2005 period with a value of 115 per 100,000 person/year (p:y). In women it continued increasing and peaked in the 2006-2010 period with an observed value of 43.3 per 100,000 p:y (Table 1 and Figure 1).

### Trend in reporting rates for the HIV/AID complex

In men there were two observed change points in the trend for reporting rates for the HIV/AIDS complex: in 1990 and 2002 (Table 2). The magnitude of the increase in reporting rates of the HIV/AIDS complex was higher during the initial phase of the epidemic, with slower growth rate of 9.8 cases per 100,000 p:y for the 1990-2002 period. There was a decreased risk of HIV/AIDS starting from this period, with an annual average change in the reporting rate of approximately 1.4 cases per 100,000 p:y (CI 95%: -4.8, 2.2) (Table 3).

**Table 2:**
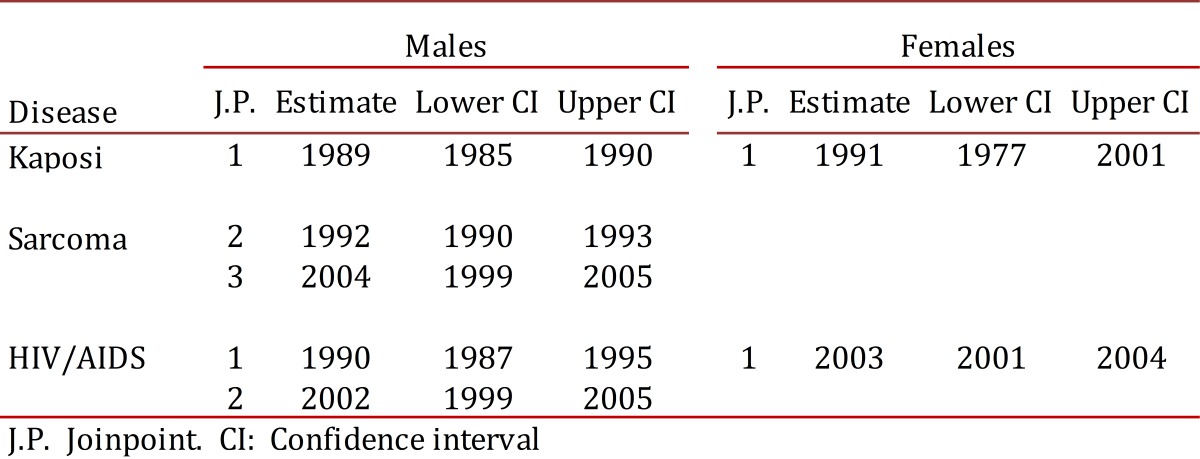
Cali, Colombia. Estimates Joinpoints in Kaposis's sarcoma incidence rates (1965-2007) and VIH/AIDS notification rates (1984-2010).

**Table 3:**
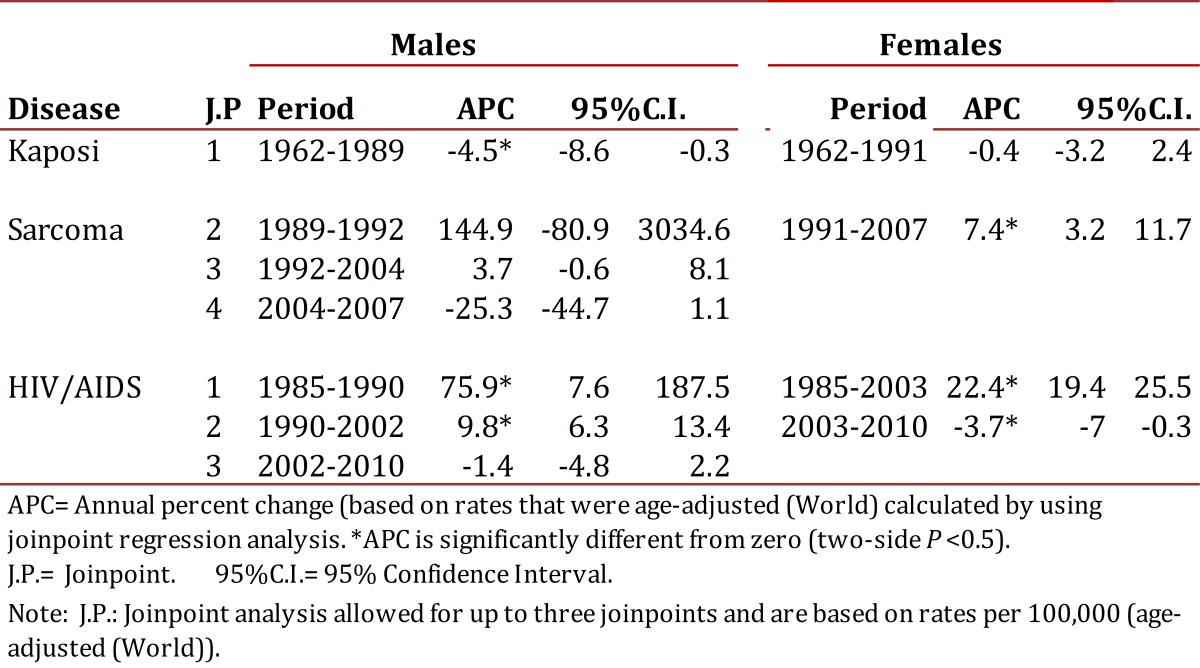
Cali, Colombia. Trends (Joinpoint analysis) in Kaposi's incidence rates (1962-2007) and HIV/AIDS notification rates (1985-2010).

Among women there was a turning point in 2003 which identified two risk periods. The average annual increase in the reporting rate until 2003 was approximately 22.4 cases per 100,000 p:y (CI 9%: 19.4, 25.5). From this peak year it significantly declined, and the average annual change in recent years was -3.7 (CI 95%: -7.0, - 0.3) (Table 3).

### Kaposi´s Sarcoma

During the period from 1963 to 2007, there were 349 new cases of KS among residents in Cali: 306 (87.7%) were males, and this type of tumor was not observed in those under 20 years of age (Table 1). The distribution of KS by location and sex varied significantly with the onset of the HIV/AIDS epidemic. During the pre epidemic period (1963-1987) only 20 cases were diagnosed with 90% of these tumors located on the skin and subcutaneous tissue and 35% occurring in women. 94.7% (329/349) of the cases of KS were diagnosed during the HIV epidemic period (1988-2007) and there were significant variations in the distribution by sex and location. The proportion of female cases was lower (10.9%, *p* <0.001). The frequency of tumors in sites other than the skin increased to 19.1% (*p* <0.01). Of the 65 cases of KS with extra cutaneous location, 27.7% occurred in the oral cavity, 16.9% in the stomach, 15.4% in the lymph nodes, 12.3% in the colon and 27.7% at other sites (small intestine, larynx, lung, bones, penis, conjunctiva, gallbladder, and kidney).

### Incidence rates for Kaposi´s Sarcoma

During the HIV epidemic, men showed an increase in the incidence rates for KS for all age groups until 2002. The risk then consistently decreased for all ages during 2003-2007. Women showed an increase in incidence rates for those over 60, but no variation in risk for those under this age (Table 1 and Figure 1).

In the five-year period following the beginning of the HIV/AIDS epidemic (i.e. 1993-1997), KS was more common in men under 40 years of age. For the period of 2003-2007 the highest risk group was those older than 60 years (Table 1).

### Incidence trends for Kaposi´s Sarcoma

**Figure 2.**
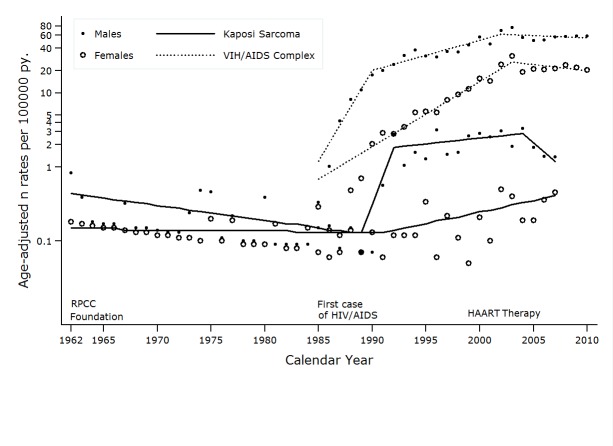
Cali, Colombia. Change in Kaposis's Sarcoma incidence rates from 1962 to 2007, three joinpoints in males (1989 1992 and 2004) and one joinpoints in females (1991). Change in VIH/AIDS notifications rates trough 1985-2010, two joinpoints in males (1990 and 2002) and one in females (2003). Rates are age adjusted to the world standard population (18 age groups).

During the period of 1965-2007, men with KS showed three change points for incidence trends: 1989, 1992 and 2004. In women, there was only one change point observed in 1991 (Table 2 and Figure 2). These change points in the incidence trend for KS pointed to four distinct periods for men and two for women with differing risks of KS (Table 3). In men the annual incidence of KS significantly decreased until 1992, then risk exponentially increased until 1995.

Following this, a slower growth rate was experienced during 1995-2007. In the last period, a clear trend toward decreasing risk of KS occurred. In women, the risk of KS was stable until 1994 and since then a significant increase in the trend rate was observed (Table 3 and Figure 2). Coinciding with the trend for incidence rates by specific ages, the regression model for segments detected a decrease in the HIV/AIDS complex global incidence rates in men and women in recent years. Particularly striking is the growing trend of KS in women, although rates are lower and less than 1 per 100,000 p:y

## Discussion

Historically, the behavior of KS in Cali, Colombia reflects its close relationship with the trend for the HIV/AIDS epidemic, contributing 94.7% of cases of sarcoma in the period from 1988 to 2007. In this, as in other studies,^10^ ^,^ ^11^ it is evident that young men remain the population group most affected by these two diseases.

The HIV/AIDS epidemic, in Cali, first began in men and the first peak in annual growth was observed around 1990. During the following decade the increase attenuated and since 2002 there is evidence of a reduction in the risk of HIV/AIDS in both sexes. The greatest cumulative frequency of HIV/AIDS cases, in Cali, occurred among men between 20 and 39 years of age. This finding is consistent with national epidemiological data, which for 2009 showed that the population group most affected by HIV/AIDS was that of men aged 25 to 34 years. There were a total of 20,573 persons affected, which corresponds to 37.8 % of the total cases reported^12^

Similar to the global and national trend^13^ the male/female ratio for HIV infection, in Cali, has a tendency to equalize, moving from 12.6 in the period 1986-1990 to 2.6 in 2006-2010. This indirectly indicates that the main route of transmission is through heterosexual relations.

In the decades that preceded the AIDS epidemic, the risk of KS in Cali remained low and stable in women, and it significantly decreased in men between 1962 and 1989, APC = -4.5% (CI 95%: -8.6, - 0.3). The magnitude, directionality and change points in the trend of reporting rates and for the KS incidence showed sex-dependent differences.

After a latency period, of almost five years after the onset of the HIV/AIDS epidemic in Cali, the increasing risk of KS manifested itself in an almost explosive manner until 1992. This coincides with the behavior of the trend in reporting rates for HIV/AIDS. Over the next 15 years the increased rate attenuated and since 2004 the risk of KS among men has shown a significant decrease. In women, the incidence rate trend for KS has been different and the magnitude of change has been less.

This discrepancy, in gender involvement, is possibly influenced by protective factors present in women that may decrease the risk of developing KS. A search began for related new pharmacological targets from observations that were carried out on pregnant women affected simultaneously by HIV and KS and who had also experienced a regression of tumor lesions. Lunardi-Iskandar et al. showed that gonadotopina chorionic (hCG), especially in the subunit β, inhibited the growth of KS cell lines in immunosuppressed mice, as did other hormones related to the menstrual cycle, such as the follicle stimulating hormone (FSH ) and luteinizing hormone (LH). However, it is known that KS tumor cells don´t have specific membrane receptors for hCG or for LH, whereas they do contain binding sites of Beta-core and hCG deglycosylated, which are metabolites derived from hCG and are apparently responsible for the described antitumor activity^14^.

In the third decade of AIDS, it is clear that the global HIV epidemic is very different from that first recognized in a small number of gay men in 1981. The epidemic has reached all countries and almost all populations of the world, most notably affecting developing countries. The remarkable advances in HIV antiretroviral therapy have led to significant changes in the survival rate and quality of life of persons with HIV, even though only one in 48 million affected people in the world has access to these medications^11^. According to a study by Hamilton *et al*., the extent of coverage by antiretroviral medications in Colombia for 2003 was estimated at 35.14%. It shows progressive improvement in the percentage of coverage with ARV, which was estimated at 49% for 2005 and 69% for 2009.

The decline in incidence rates of KS among males in Cali coincides in time with the introduction of HAART in Colombia. However, this effect is now evident a decade after it was first reported in developed countries. This gap between different developing countries suggests that there is a clear delay in the transfer of technological advances. The local risk reduction of KS and HIV/AIDS in men during the last five-year evaluation speaks indirectly of factors that prevent infection by both agents or restrict their development. The phenomenon is probably influenced largely by the extent of promotion and prevention programs, responsible sexual behavior, and by increased coverage of antiretroviral medication. It is also possible that cases of HIV/AIDS are underreported.

This favorable change in the KS incidence trend is still not evident among women in Cali. In contrast, a significantly increased risk of KS during the period 1991-2007 was observed, with an average annual change of 7.4% (CI 95%: 3.2-11.7). This discrepancy in the response could be related to the unequal access to medical care and to the use of HAART.^11^ ^,^ ^15^ In addition, women tend to have lower adherence to antiretroviral therapy, which possibly occurs from experiencing a greater number of adverse effects during treatment with HAART.^16^ The reasons for these gender differences in adverse drug effects are not clear. ^17^ ^,^ ^18^

UNAIDS and WHO claim that the global HIV incidence peaked sometime late in the last decade, when nearly three million people in a single year were infected. Since then, the number of new infections per year has slowly declined reflecting both the natural course of the global epidemic and the success of HIV prevention efforts^19^.

During the global HIV epidemic, difficulty in interpreting the trends in malignancies associated with HIV has been due to the lack of appropriate information. Cancer registries are one resource for timely population-based surveillance of HIV-associated malignancies, but it also has limitations for continuous monitoring. Although 85-90% of HIV-associated malignancies are detected, they are currently ill-equipped for follow-up because the status of the HIV infection is not systematically collected. Registries for AIDS only collect information on AIDS-defining cancers (KS, NHL, cervical cancer) and do not detect cases that develop after the diagnosis of AIDS.

The most comprehensive studies of temporary and absolute associations with AIDS and cancer have involved pairing of data bases and population-based cancer registries and registries for AIDS. However, these pairings are not performed routinely, and little is known about their quality in populations that use HAART, for which AIDS is a poor substitute for the HIV-positive status. An alternative resource for the study of HIV-associated cancers is the population-based HIV registry which has been used successfully in Australia. However, in many Western countries, the usefulness of these records, used to establish parings with cancer databases, may be limited by confidentiality practices because they exclude the collection of patient identities.^20^

The effect of HAART therapy on the full spectrum of cancers associated with HIV infection is still unknown. If HIV treatment is successful in restoring the immune system, then the incidence of some cancers may be reduced or at least delayed. However, reduction of morbidity from HIV infection and increased life expectancy associated with HAART therapy may provide the required latency period for the development of other types of cancer, including those not previously identified as HIV related. A recent review found that the incidence of solid tumors and hematologic malignancies is higher in HIV-infected patients in comparison with the general population. Consequently, it will be important to study cancer incidence data from HIV-infected persons over time to know the true impact of the HAART therapy.^21^

It is necessary to evaluate other defining and non-defining malignant cancers arising from AIDS in this population group to define the magnitude of the problem and devise preventive strategies.
